# Calorie Restriction in Adulthood Reduces Hepatic Disorders Induced by Transient Postnatal Overfeeding in Mice

**DOI:** 10.3390/nu11112796

**Published:** 2019-11-16

**Authors:** Catherine Yzydorczyk, Na Li, Eve Rigal, Hassib Chehade, Dolores Mosig, Jean Baptiste Armengaud, Thibaud. Rolle, Anithan Krishnasamy, Eulalia Orozco, Benazir Siddeek, Christian Juvet, Catherine Vergely, Umberto Simeoni

**Affiliations:** 1 DOHaD Laboratory, Woman-Mother-Child Department, Division of Pediatrics, Centre Hospitalier Universitaire Vaudois and University of Lausanne, 1011 Lausanne, Switzerland; hassib.chehade@chuv.ch (H.C.); dolores.mosig@me.com (D.M.); jean-baptiste.armengaud@chuv.ch (J.B.A.); Rotib7@gmail.com (T.R.); anithan.krishnasamy@gmail.com (A.K.); eulalia_of@hotmail.com (E.O.); benazir.siddeek@chuv.ch (B.S.); christian.juvet@chuv.ch (C.J.); umberto.simeoni@chuv.ch (U.S.); 2 Equipe Physiopathologie et Epidémiologie Cérébro-Cardiovasculaires (PEC2, EA7460), UFR Sciences de Santé, Université de Bourgogne Franche-Comté, 21000 Dijon, France; na.li@unil.ch (N.L.); cvergely@u-bourgogne.fr (C.V.)

**Keywords:** liver, developmental programming, stress-induced premature senescence, oxidative stress, reversibility, DOHaD

## Abstract

Impaired early nutrition influences the risk of developing metabolic disorders in later life. We observed that transient postnatal overfeeding (OF) in mice induces long-term hepatic alterations, characterized by microsteatosis, fibrosis associated with oxidative stress (OS), and stress-induced premature senescence (SIPS). In this study, we investigated whether such changes can be reversed by moderate calorie restriction (CR). C57BL/6 male mice pups were maintained during lactation in litters adjusted to nine pups in the normal feeding (NF) group and three pups in the transient postnatal OF group. At six months of age, adult mice from the NF and OF groups were randomly assigned to an *ad libitum* diet or CR (daily energy supply reduced by 20%) for one month. In each group, at the age of seven months, analysis of liver structure, liver markers of OS (superoxide anion, antioxidant defenses), and SIPS (lipofuscin, p53, p21, p16, pRb/Rb, Acp53, sirtuin-1) were performed. CR in the OF group reduced microsteatosis, decreased levels of superoxide anion, and increased protein expression of catalase and superoxide dismutase. Moreover, CR decreased lipofuscin staining, p21, p53, Acp53, and p16 but increased pRb/Rb and sirtuin-1 protein expression. CR did not affect the NF group. These results suggest that CR reduces hepatic disorders induced by OF.

## 1. Introduction

In recent years, the prevalence of obesity has dramatically increased worldwide and is considered a major global health problem. Notably, obesity in children/adolescents is particularly worrying. In 40 years, the number of school-age children and adolescents with obesity has greatly increased from 11 million to 124 million [1]. Adverse health consequences of obesity are observed in many western populations and include higher blood pressure, obesity, glucose intolerance, dyslipidemia, decreased insulin sensitivity, and type 2 diabetes, which are components of metabolic syndrome (MetS) [2]. Nonalcoholic fatty liver disease (NAFLD), which is the major prevalent acquired chronic liver disease in developed countries [3], is considered a hepatic manifestation of MetS [4]. Impaired nutritional conditions during early development, such as maternal and paternal undernutrition or overnutrition, maternal dietary imbalance during pregnancy, and altered early postnatal nutrition, are associated with the developmental programming of cardiometabolic diseases in adulthood [2].

Such clinical and epidemiological observations have been reproduced in animal models. In rodents, reduced litter size after birth decreases competition for milk during the lactation period and therefore induces overnutrition in the early postnatal period. At weaning, increased body weight and fat content have been observed [5]. Furthermore, in adulthood, early postnatal overfeeding (OF) induces metabolic and cardiovascular alterations [6,7,8,9,10,11].

The liver plays a key role in lipid and glucose metabolism. Liver function and structure are particularly sensitive to oxidative stress (OS)-related damage that can lead to hepatocyte dysfunction, notably accelerated senescence, which is involved in acute and chronic liver disease [12].

We have previously observed that transient postnatal OF during the suckling period in mice led to liver microsteatosis and fibrosis in adulthood, and this outcome was associated with OS and stress-induced premature senescence (SIPS) [13]. Calorie restriction (CR), as a potential preventive strategy for metabolic disorders, has received considerable interest. CR, which can be defined as a decrease in energy intake without malnutrition, has been shown to be protective against liver diseases in human studies [14,15] and to improve health span, delay cardiac aging, and improve cardiometabolic health [16,17]. In animal models, CR reduced NAFLD in db/db mice [18] and middle-aged C57BL/6J mice [19] and reversed glucose metabolism and cardiac dysfunctions induced by transient postnatal OF [11]. A proposed explanation for these beneficial effects is that CR results in a mildly stressful situation that elicits a survival response, notably by modulating sirtuin-1 (Sirt-1) activity and expression [20], which helps the organism survive in adverse situations by altering the metabolism and increasing defenses against the causes of aging, whatever the origins may be [21].

However, whether CR in adulthood can reverse hepatic alterations programmed by altered early nutrition is still unknown. In this study, we explored whether moderate CR (20%) over a short period (one month) in adulthood can prevent hepatic structure alterations, liver OS, and SIPS induced by transient postnatal OF in a mouse model.

## 2. Materials and Methods

### 2.1. Animal Model

Investigations were performed in accordance with the Directive 2010/63/EU of the European Parliament and the Guidelines for the Care and Use of Laboratory Animals published by the U.S. National Institutes of Health (NIH Publication No. 85–23, revised 1996). The Comité d’Ethique de l’Experimentation Animale, Université de Bourgogne-Franche-Comté, Dijon, France acted as the institutional review board (protocol agreement number: 00412.03) and specifically approved this study.

In this study, we used the same cohort of animals that was previously described by Li et al. [11]. Briefly, adult female C57BL/6 mice (Charles River, L’Arbresle, France) at six weeks of age were individually housed. After one week of adaptation, they were mated overnight with males at a proportion of 2:1. On the third day of life, male pups were randomly distributed among mothers to achieve cross fostering. Litter sizes were adjusted to either nine male pups in the normal feeding (NF) group or three male pups in the postnatal overfeeding (OF) group during the lactation period (three weeks). Each litter included pups from one to six different dams to increase genetic variability within the litters. After weaning, the mice in both groups had free access to a standard diet (A04, SAFE Diets Augy, France) and water.

At six months of life, mice were randomly assigned for one month to either an *ad libitum* diet (NF and OF groups) or to a CR diet induced by a reduction in daily food supply of 20% (based on the food intake of each group), which led to the establishment of the NFCR and OFCR groups. To perform all the experiments presented in this study, two consecutive series of reproductions were used, corresponding to 12 different litters.

Only males from each group (NF (*n* = 5–6), OF (*n* = 5–6), NFCR (*n* = 4–5), and OFCR (*n* = 4–5)) were studied at seven months of age. After 6 h of fasting, mice were sacrificed, and the livers were then harvested and immediately frozen in nitrogen for western blot analyses or fixed in formol for histological analyses.

### 2.2. Evaluation of Hepatic Structure by Histological Analysis

At seven months of life, the livers from the NF (*n* = 5), OF (*n* = 5), NFCR (*n* = 5) and OFCR (*n* = 5) groups were rapidly removed and fixed in formol as previously described [13]. Equatorial cross-sections were paraffin-embedded and stained with hematoxylin and eosin (H/E) for hepatic structure evaluation and with Masson’s Trichrome to evaluate hepatic fibrosis. For all histological analyses, the slides were observed blindly by the same experimenter (C.Y), and three images were captured for each animal. A pathologist (Prof. C. Sempoux) confirmed the histological observations. Hepatic fibrosis was quantified using ImageJ 1.50b (Java 1.8.0_60, National Institutes of Health, MD, USA) (http://rsbweb.nih.gov/ij), as previously described [13].

### 2.3. Detection of Superoxide Anion (O_2_^•−^) by Chemiluminescence

Liver O_2_^•−^ production was evaluated at seven months of life in the NF (*n* = 5), OF (*n* = 5), NFCR (*n* = 5), and OFCR (*n* = 5) groups using oxidative fluorescent dye hydroethidine (2 μM, Sigma-Aldrich) [22,23] as previously described [13]. Briefly, liver sections were stained with hydroethidine by incubating the sections in a light-protected humidified chamber at 37 °C for 30 min. The sections were rinsed with phosphate-buffered saline (PBS) and mounted using Fluoromount-G mounting medium with 4′6-diamidino-2-phenylindole (DAPI; Interchim, France). The slides were observed blindly by the same experimenter (C.Y), and images were obtained using a laser scanning confocal microscope (Leica SP5) equipped with an argon laser. At least four hepatic sections were assessed per animal with a 514-nm long-pass filter and were evaluated with ImageJ software [13].

### 2.4. Detection of Oxidative DNA Double-Strand Break

As previously described [13], liver sections from 7-month-old mice from the NF (*n* = 5), OF (*n* = 5), NFCR (*n* = 5) and OFCR (*n* = 5) groups were stained with 53BP-1 (1/100, Abcam, #ab21083) overnight at 4 °C. The sections were then washed with PBS and incubated for two hours with Alexa Fluor-488-conjugated donkey anti-rabbit IgG (1/200, Abcam, #ab150073). The sections were then rinsed with PBS and mounted using Fluoromount-G mounting medium with DAPI. A negative control was obtained by incubation only with secondary antibody. The slides were observed blindly using a fluorescence microscope (Nikon, Eclipse Ti2 Series) by the same experimenter (C.Y), and at least four hepatic sections were assessed per animal using ImageJ software [13].

### 2.5. Detection of Liver Senescence by Histological Staining

A marker of SIPS [24], lipofuscin, was identified by diastase periodic acid Schiff (d-PAS) resistance and Fontana Masson and Sudan Black B (SBB) staining of livers from NF (*n* = 5), OF (*n* = 5), NFCR (*n* = 5), and OFCR (*n* = 5) groups. For all histological analyses, three images were captured for each animal. Lipofuscin staining was quantified using ImageJ software. A quantitative analysis was performed blindly by a single experimenter (C. Y), as previously described [13].

### 2.6. Western Blotting

Liver proteins from the NF (*n* = 5), OF (*n* = 5), NFCR (*n* = 4) and OFCR (*n* = 4–6) groups were extracted at seven months of life (from the medial lobe of the snap-frozen livers) using radioimmunoprecipitation assay buffer (RIPA) buffer as previously reported [13]. Proteins were quantified (Pierce BCA Protein Assay Kit, Thermo Scientific, Rockford, IL, USA) and western blotting was performed. Denatured liver proteins (30 μg) from the specific groups were separated using gradient gel (NuPAGE 4–12% Bis-Tris gel, Thermo Scientific) and transferred overnight at 4 °C. All primary antibody incubations were performed in blocking buffer tris-buffered saline ((TBS)-Tween 2%-bovine serum albumin (BSA) 3%) overnight at 4 °C. Antibodies (Cell Signaling 1/1000e) against catalase (Cat; #14097), Sirt-1 (#9475), retinoblastoma tumor suppressor protein (Rb, #9313) and phospho-Rb (pRb, Ser807/811, #8516); p53 (#2524) and acetyl-p53 (Lys379, #2570); p21^WAF^, CDKN2A/p16^INK4a^, and Cu/Zn superoxide dismutase (Sod) (Abcam, 1/1000e, #ab188224; # ab201980 and #ab13498, respectively); and alpha smooth muscle actin (a-SMA, Sigma-Aldrich, 1/1000e, #A5228) were purchased. Incubations were performed in blocking buffer at room temperature for one hour with anti-mouse or anti-rabbit secondary antibodies (1/2000; Cell Signaling, #7076 and #7074, respectively). The antibodies were visualized using enhanced chemiluminescence western blotting substrate (Thermo Scientific). To detect specific bands, a G-BOX Imaging System (GeneSys, Syngene, Cambridge, UK) or a photographic film (CL-XPosureTM Film, Thermo Scientific) was used, and the optical density of each band was scanned and measured using specific software (GeneTools 4.03.05.0, Cambridge, England). Full-length western blots are presented.

### 2.7. Statistical Analyses

For each parameter, the mean ± standard deviation (SD) was used for comparisons between groups. Given the small sample size, the means of each parameter of interest were compared using a nonparametric Mann-Whitney U test. The significance level was set at *p* < 0.05. Statistical analyses were performed and graphics were created using GraphPad Prism version 8.3.0 (La Jolla, CA, USA).

## 3. Results

### 3.1. Effect of Calorie Restriction on Liver Structure

Hepatic steatosis was evaluated by H/E, and hepatic fibrosis was explored with Masson’s Trichrome staining and with the measurement of α-SMA protein expression in the NF, NFCR, OF, and OFCR groups.

**Figure 1:**
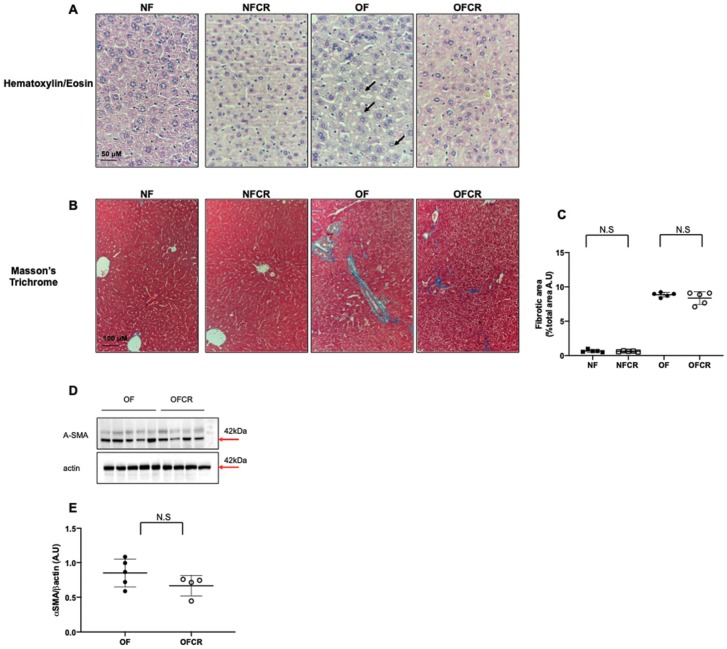
Hepatic structure of the animals in the normal feeding (NF), normal feeding calorie restriction (NFCR), overfeeding (OF), and overfeeding calorie restriction (OFCR) groups at seven months of age. Basic liver histology was evaluated using hematoxylin (nuclear localization) and eosin (cytoplasmic localization) staining (**A**) in the NF, NFCR, OF, and OFCR groups at 40× to evaluate steatosis. The arrows indicate slight microsteatosis in the OF group. Hepatic fibrosis was evaluated with Masson’s Trichrome staining (**B**) in the NF, NFCR, OF and OFCR groups at 20× and was quantified using ImageJ (**C**). N.S: nonsignificant, *p* > 0.05. These pictures are representative images from *n* = 5 animals/group. In (**D**,**E**), alpha-SMA protein expression was measured using western blot in the OF and OFCR groups. Values are reported as the mean ± SD; N.S nonsignificant, *p* > 0.05; *n* = 5–4. animals/group.

In the livers from the OFCR group compared to those of the OF group, we observed, using H/E, a decreased wispy cleared cytoplasm indicating reduced microsteatosis (Figure 1A). In the livers from the NFCR and NF groups, we observed no lipid droplet accumulation via H/E staining (Figure 1A).

With Masson’s Trichrome staining, we observed that the mean percentage of hepatic fibrotic area was not significantly different between the livers from the OFCR group and the livers from the OF group (OFCR vs. OF (arbitrary units (A.U.) ± SD) 7.98 ± 1.24 vs. 9.04 ± 0.55; *p* > 0.05) (Figure 1B,C). Additionally, we observed no significant difference in α-SMA protein expression between the OFCR and OF groups (OFCR vs. OF (A.U. ± SD) 0.66 ± 0.07 vs. 0.85 ± 0.09; *p* > 0.05) (Figure 1D,E). No difference was observed between the livers from the NFCR group and those from the NF group (NFCR vs. NF (A.U. ± SD) 0.66 ± 0.17 vs. 0.70 ± 0.20; *p* < 0.001; Figure 1B,C).

### 3.2. Effect of Calorie Restriction on Oxidative Stress in Hepatic Tissue

**Figure 2:**
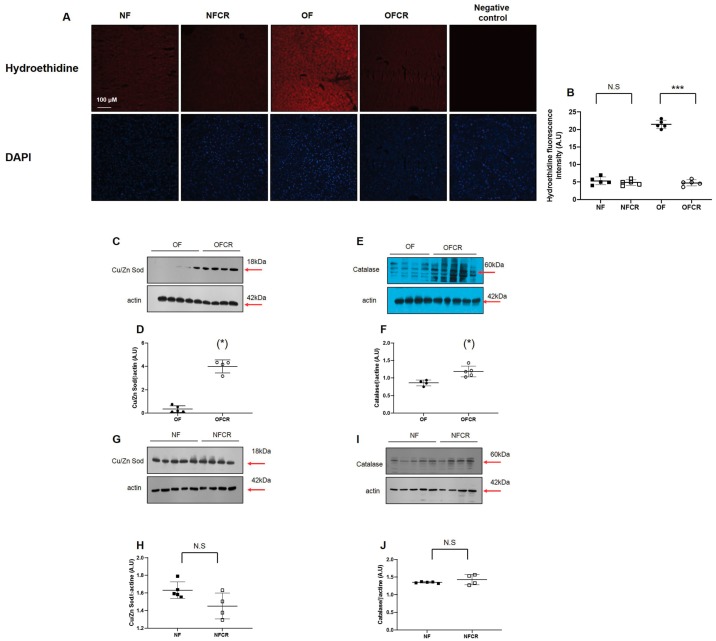
Liver superoxide anion production and antioxidant defense protein expression in the NF, NFCR, OF, and OFCR groups at seven months of age. Superoxide anion production was measured by hydroethidine staining (**A**) at the same magnification (20×) in the NF, NFCR, OF, and OFCR groups. Nuclei were counterstained with DAPI. A negative control was included. Hydroethidine fluorescence intensity was quantified using ImageJ (**B**). ***p < 0.001, N.S: nonsignificant, *p* > 0.05. These pictures are representative images from *n* = 5 animals/group. Cu/Zn superoxide dismutase (Cu/Zn Sod) (**C**,**D**) and catalase (**E**,**F**) protein expression levels were measured by western blot in the OF and OFCR groups. Cu/Zn superoxide dismutase (Cu/Zn Sod) (**G**,**H**) and catalase (**I**,**J**) protein expression levels were also measured by western blot and in the NF and NFCR groups. Values are reported as the mean ± SD; (*) *p* < 0.05; N.S: nonsignificant, *p* > 0.05; *n* = 5–4. animals/group.

Liver OS was investigated by superoxide anion production (hydroethidine) quantification and catalase and Cu/Zn Sod protein expression measurement. The livers from the OFCR group, compared to OF group, displayed significantly decreased O_2_^•−^ production (OFCR vs. OF (A.U. ± SD) 4.75 ± 0.84 vs. 21.45 ± 1.18; *p* < 0.001) (Figure 2A,B).

We observed an increase in Cu/Zn Sod (+564%; *p* < 0.05) (Figure 2C,D) and catalase (+38%; *p* < 0.05) (Figure 2E,F) protein expression in the livers from the OFCR group compared to that in the livers of the OF group.

No difference was observed between the NFCR and NF groups in regard to O_2_^•−^ production (NFCR vs. NF (A.U. ± SD) 4.83 ± 0.79 vs. 5.33 ± 1.12; *p* > 0.05) (Figure 2A,B) or in regard to Cu/Zn Sod (Figure 2G,H) and catalase (Figure 2I,J) protein expression.

### 3.3. Effect of Calorie Restriction on DNA Double-Strand Break in Hepatic Tissue

**Figure 3:**
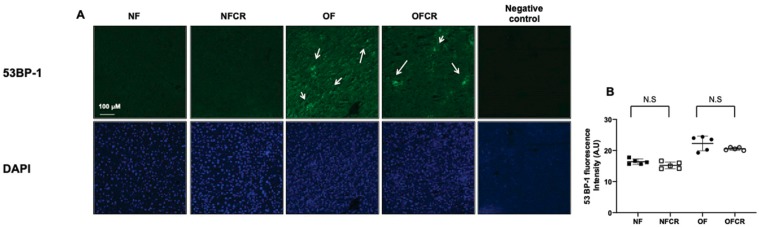
Liver DNA double-strand break evaluation in the NF, NFCR, OF, and OFCR groups at seven months of age. DNA double-strand breaks were evaluated by 53BP-1 staining at the same magnification (20×) in the NF, NFCR, OF, and OFCR groups (**A**). The presence of a DNA double stand break was observed in the OF and OFCR groups (arrows). Nuclei were counterstained with DAPI. A negative control was included. These pictures are representative images from *n* = 5 animals/group. 53BP-1 fluorescence intensity was quantified using ImageJ (**B**), N.S: nonsignificant, *p* > 0.05.

The DNA double-strand breaks were explored using 53BP-1 staining. The livers from the OFCR group displayed no significant decrease in 53BP-1 staining compared to those from the OF group (OFCR vs. OF (arbitrary units ± SD) 21.41 ± 2.88 vs. 20.62 ± 4.88; *p* > 0.05) (Figure 3A,B).

No difference was observed between the NFCR and NF groups concerning 53BP-1 staining (NFCR vs. NF (A.U. ± SD) 16.21 ± 2.07 vs. 16.55 ± 1.05; *p* > 0.05) (Figure 3A,B).

### 3.4. Effect of Calorie Restriction on Histological Detection of Senescence

**Figure 4:**
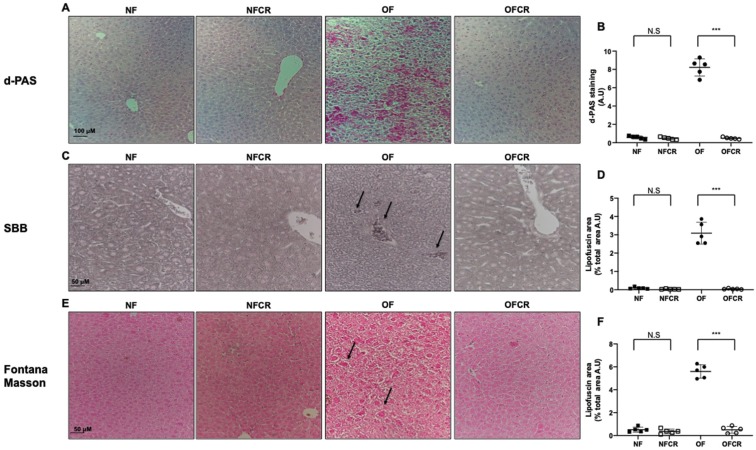
Lipofuscin accumulation using diastase periodic-Schiff (d-PAS), Sudan black B (SBB) and Fontana Masson staining in the livers of the animals in the NF, NFCR, OF and OFCR groups at 7 seven months of age. d-PAS staining (**A**) was evaluated in the NF, NFCR, OF and OFCR groups at 20×. d-PAS staining was quantified using ImageJ (**B**), *** *p* < 0.001; N.S: nonsignificant, *p* > 0.05. SBB staining (**C**) (arrow in the OF group) was evaluated in the NF, NFCR, OF and OFCR groups at 40×. SBB staining was quantified using ImageJ (**D**), *** *p* < 0.001; N.S: nonsignificant, *p* > 0.05. Fontana Masson staining (**E**) (arrow in OF group) was evaluated in the NF, NFCR, OF and OFCR groups at 40×. Fontana Masson staining was quantified using ImageJ (**F**), *** *p* < 0.001; N. S: nonsignificant, *p* > 0.05. These pictures are representative images from *n* = 5 animals/group.

The presence of lipofuscin deposits was detected using d-PAS resistance, SBB and Fontana Masson staining. The livers from the OFCR group, compared to OF group, displayed decreased d-PAS (OFCR vs. OF (A.U. ± SD) 0.48 ± 0.11 vs. 8.22 ± 0.94; *p* < 0.001) (Figure 4A,B), SBB (OFCR vs. OF (A.U. ± SD) 0.50 ± 0.13 vs. 2.88 ± 0.15; *p* < 0.001) (Figure 4C,D) and Fontana Masson (OFCR vs. OF (A.U. ± SD) 1.89 ± 0.20 vs. 6.92 ± 0.22; *p* < 0.001) (Figure 4E,F) staining.

No difference between the livers of the NFCR group and those of the NF group was observed concerning d-PAS (NFCR vs. NF (A.U. ± SD) 0.47 ± 0.13 vs. 0.57 ± 0.13; *p* > 0.05) (Figure 4A,B), SBB (NFCR vs. NF (A.U. ± SD) 0.47 ± 0.11 vs. 0.57 ± 0.13; *p* > 0.05) (Figure 4C,D), or Fontana Masson (NFCR vs. NF (A.U. ± SD) 1.69 ± 0.10 vs. 1.80 ± 0.06; *p* > 0.05) (Figure 4E,F) staining.

### 3.5. Effect of Calorie Restriction on Molecular Pathways of Senescence in Hepatic Tissue

**Figure 5:**
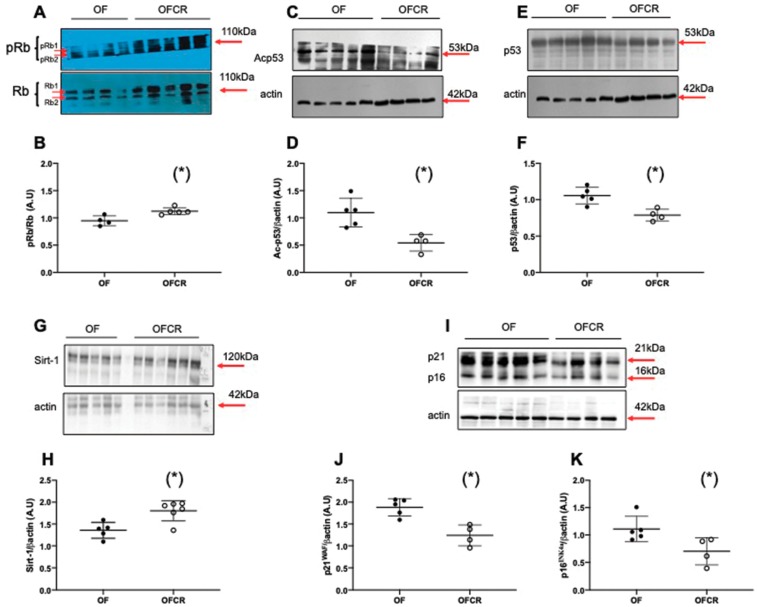
Hepatic senescence in the animals in the OF and OFCR groups at seven months. Liver protein levels of pRb/Rb (**A**,**B**), Ac-p53 (**C**,**D**), p53 (**E**,**F**), Sirt-1 (**G**,**H**), p21^WAF^ (**I**,**J**) and p16^INK4a^ (**I**,**K**) were measured by western blot in the OF and OFCR groups. Values are reported as the mean ± SD; (*) *p* < 0.05; *n* = 5–4 or 6 animals/group.

Molecular senescence was investigated by measuring pRb/Rb, Ac-p53, p53, p21^WAF^, p16 ^INK4a^, and Sirt-1 protein levels. The livers from the OFCR group displayed a significant decrease in Acp53 (−49%; *p* < 0.05) (Figure 5C,D), p53 (−23%; *p* < 0.05) (Figure 5E,F), p21^WAF^ (−35%; *p* < 0.05) (Figure 5I,J), and p16^INK4a^ (−31%; *p* < 0.05) (Figure 5I,K) protein expression and a significant increase in the protein expression of pRb/Rb (+20%; *p* < 0.05) (Figure 5A,B) and Sirt-1 (+24%; *p* < 0.05) (Figure 5G,H).

**Figure 6:**
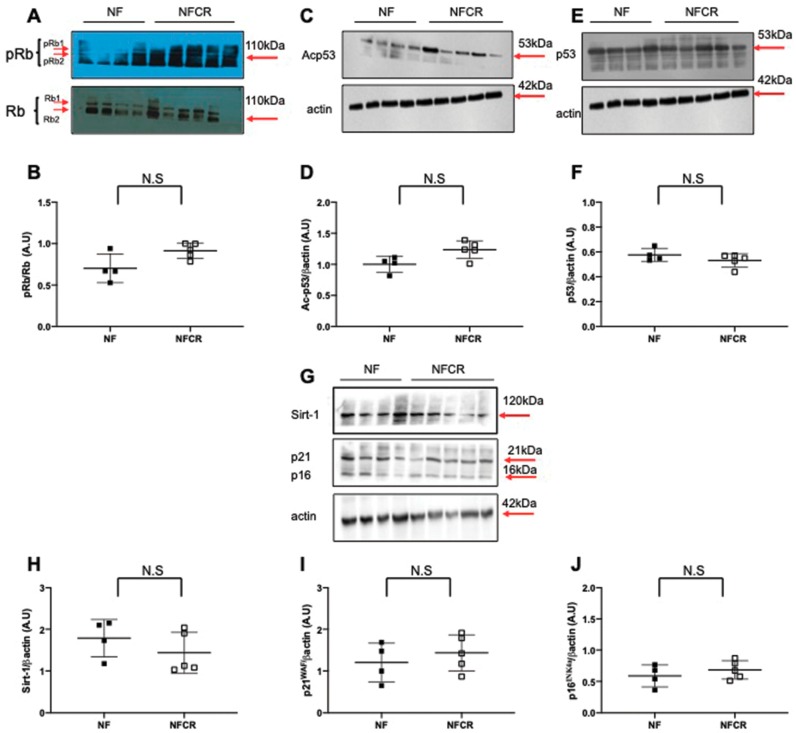
Hepatic senescence in animals in the NF and NFCR groups at seven months. Liver protein levels of p-Rb/Rb (**A**,**B**), Ac-p53 (**C**,**D**), p53 (**E**,**F**), Sirt-1 (**G**,**H**), p21^WAF^ (**G**,**I**) and p16^INK4a^ (**G**,**J**) were measured by western blot in the NF and NFCR groups. Values are reported as the mean ± SD; N.S: nonsignificant, *p* > 0.05; *n* = 5–4 animals/group).

We observed no significant differences in pRb/Rb (Figure 6A,B, *p* > 0.05), Ac-p53 (Figure 6C,D, *p* > 0.05), p53 (Figure 6E,F, *p* > 0.05), Sirt-1 (Figure 6G,H, *p* > 0.05), p21^WAF^ (Figure 6G,I, *p* > 0.05) or p16^INK4a^ (Figure 6G,J, *p* > 0.05) protein expression in the livers from the NFCR group compared to those in the NF group.

## 4. Discussion

In this study, moderate CR in adulthood reversed microsteatosis, liver OS, and SIPS induced by transient postnatal OF in mice.

We explored the effects of CR on hepatic structure, particularly on liver microsteatosis and fibrosis induced by transient postnatal OF. Using H/E staining, we observed, as shown in Figure 1A, that CR only in the livers from 7-months-old mice reduces hepatic microsteatosis with decreased in wispy cleared cytoplasm characterized by ballooned cells, as previously observed in db/db mice [18]. Hepatic steatosis is a feature related to obesity [25] and impaired insulin sensitivity [26]. Therefore, this reduction of microsteatosis by CR could be the consequence of decreased body weight and improved insulin sensitivity and glucose tolerance, as observed previously by Li et al. in the same animal model that we used in this study [11]. Furthermore, we observed in the livers of 7-month-old OF mice that CR did not significantly decrease collagen accumulation, identified by Masson’s Trichrome staining, or alpha-SMA protein expression, as shown in Figure 1B,D, so CR demonstrated no significant effect on hepatic fibrosis, which is contrary to the findings of other studies [18]. This difference may be due to the moderate (20%) and shorter (one month) CR used in our study. In addition, hepatic fibrosis is commonly considered an irreversible scarring of liver tissue with an excessive presence of extracellular matrix. CR had no effect on hepatic structure in the NF group.

The liver is particularly sensitive to OS-related damage, which can lead to the development of hepatic disorders. Increased liver reactive oxygen species (ROS) production can occur due to an imbalance between an increased production of ROS and a decreased level of antioxidant defenses, especially ROS scavengers. CR is among the therapies proposed to decrease OS [27,28]. We observed in the livers of 7-month-old OF mice that CR decreased superoxide anion levels, as shown in Figure 2A, and increased catalase and, particularly, Cu/Zn Sod protein expression, as shown in Figure 2E,C, respectively. It has been shown that CR improves lifespan via H_2_O_2_ production and by increasing the activity and expression of Cu/Zn Sod [29,30]. These results suggest that CR decreased the liver OS induced by transient postnatal OF by improving endogenous antioxidant defense capability. Similar results have been described in regard to livers from rats exposed to dietary restriction (40% restriction of energy intake) [31]. In the NF group, we observed no effects of CR on superoxide anion production, as shown in Figure 2A, or Cu/Zn Sod or catalase protein expression, as shown in Figure 2G,I, respectively.

Increased ROS production can induce DNA damage [32]. The protein 53BP-1 is implicated in double-strand break repair, and the levels of 53BP-1 are elevated in many DNA damage response conditions [33]. CR can increase the level of repair of damaged macromolecules [34]. However, CR had no significant effect on 53BP-1 staining in the livers of 7-month-old OF mice, as shown in Figure 3, suggesting that in our animal model, CR could not repair the DNA double-strand breaks induced by postnatal OF. A possible explanation is that the repair of the DNA damage induced by OS (increased superoxide anion production and decreased catalase and Cu/Zn superoxide dismutase protein expression) would be lower in calorie-restricted animals due to the lower rates of free radical attack on DNA [35]. CR had no effect on 53BP-1 staining in the NF group in Figure 3.

The accumulation of molecular oxidative damage in various components of the cell that can alter physiological functions is in agreement with the “Oxidative Stress Theory of Aging” [36]. Cellular senescence, which is defined as a sustained antiproliferative response arresting the cell cycle, has been related to increased ROS levels and DNA double-strand breaks [13,37,38]. CR can slow down the aging process [39]. We evaluated the effect of CR on cellular senescence using the detection of lipofuscin, which is an aggregate of oxidized proteins that accumulate progressively, mostly in aged postmitotic cells [40]. Lipofuscin has been identified as a cellular senescence biomarker, particularly a marker of SIPS. We detected lipofuscin accumulation using SBB, Fontana Masson, and d-PAS-resistant staining. We also measured the protein expression of several factors associated with senescence, such as p53, p21^WAF^, p16^INK4a^, and retinoblastoma (Rb) phosphorylation. Among these factors, the induction of p16^INK4a^ and p53 expression appears to be associated with premature senescence [41]. We observed in the livers from 7-month-old OF mice that CR decreased d-PAS (Figure 4A), SBB (Figure 4C), and Fontana Masson (Figure 4E) staining. Therefore, CR reversed lipofuscin accumulation induced by transient postnatal OF. Moreover, we observed that CR decreased p53, p21^WAF^, and p16^INK4a^ and increased pRb/Rb expression in the livers of 7-month-old OF mice, and these expression levels were up- and downregulated, respectively, by transient postnatal OF (Figure 5). In other models of CR in mice, decreased liver histological senescence biomarkers and p16^INK4a^ expression have also been reported [42,43]. We observed no effect of CR in the NF group on lipofuscin staining (Figure 4) or on p53, p21^WAF^, p16^INK4a^ or pRb/Rb protein expression (Figure 6).

Additionally, a family of deacylase proteins, the sirtuins, has been identified as regulating the senescence process [44]. In particular, Sirt-1 is a nutrient sensor that regulates the expression of several genes involved notably in stress tolerance by modulating antioxidant defenses [45], fat metabolism, and the regulation of longevity [46]; notably these modulations occur due to its modulation by CR [47]. Moreover, a relationship has been observed among CR, Sirt-1, p16^INK4a^, and senescence. In human cells, Sirt-1, due to its activation by CR, directly binds to the p16^INK4a^ promoter, which decreases its expression through a deacylation effect of Sirt-1 and contributes to delaying the aging process and therefore to extending lifespan [48]. We observed in the livers of 7-month-old OF mice that CR improved Sirt-1 protein expression (Figure 5G) and activity by decreasing the acylation of p53 at Lys-379 (Figure 5C), which could explain the higher catalase and Cu/Zn Sod protein expression, leading to the reduction in the liver superoxide anion levels, as observed in Figure 2. Additionally, increased expression and restored deacylase activity of Sirt-1 induced by CR could also explain the reduction of hepatic microsteatosis and obesity induced by transient postnatal OF. In fact, overexpression of Sirt-1 in mice exposed to a high-fat diet provided protection against hepatic steatosis [49,50] and obesity [51,52]. We observed no effect of CR on Sirt-1 protein expression in the NF group, as shown in Figure 6G.

This study has some limitations. The animals were studied on the last day of CR; therefore, the persistence of the beneficial effects induced by CR when mice once again received an ad libitum diet is unknown. No serum/plasma samples were available to measure the ALAT and ASAT expression as well as pro-inflammatory cytokines. Only males were included in this study, and the characterization of an eventual sexual dimorphism will need further investigation. Further studies are required to identify early biomarkers of these hepatic disorders, notably using epigenetic tools such as DNA methylation and miRNAs.

## 5. Conclusions

**Figure 7:**
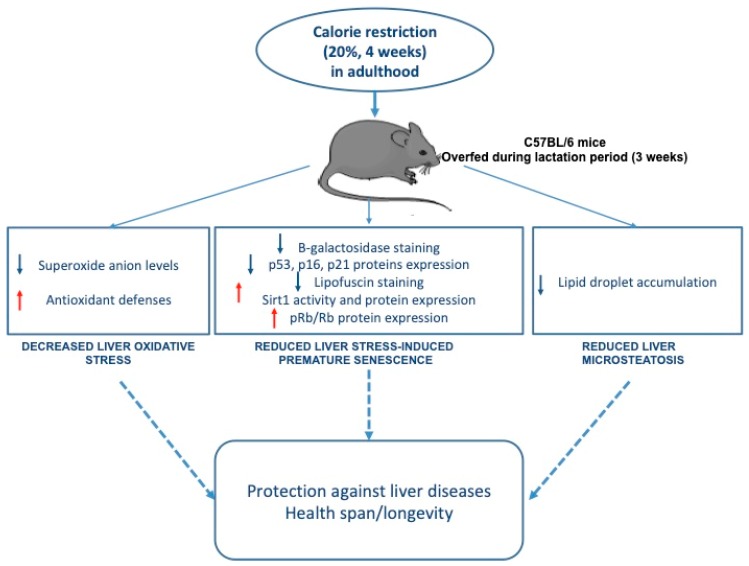
The effects of moderate calorie restriction in adulthood on liver dysfunction induced by transient postnatal overfeeding in C57BL/6 mice.

In conclusion, this study demonstrates that moderate CR in adulthood reduces liver microsteatosis, OS, and SIPS induced by transient postnatal OF during the lactation period (Figure 7). Therefore, even late CR can confer a protective effect against liver disease. This study also demonstrates that an appropriate eating behavior in adulthood can reduce the long-term effects of altered early nutrition.

## Abbreviations

α-SMA: alpha smooth muscle actin; CR: calorie restriction; Cu/Zn Sod: Cu/Zn superoxide dismutase; d-PAS: diastase periodic acid Schiff; H/E: hematoxylin/eosin; MetS: metabolic syndrome; NAFLD: nonalcoholic fatty liver disease; NF: normal feeding; NFCR: normal feeding calorie restriction; OF: overfeeding; OFCR: overfeeding calorie restriction; OS: oxidative stress; SBB: Sudan black B; SD: standard deviation; SIPS: stress-induced premature senescence; Sirt-1: sirtuin-1; (p)Rb: (phospho) retinoblastoma tumor suppressor.
